# Home support workers perceptions of family members of their older clients: a qualitative study

**DOI:** 10.1186/s12877-015-0163-4

**Published:** 2015-12-12

**Authors:** Joanie Sims-Gould, Kerry Byrne, Catherine Tong, Anne Martin-Matthews

**Affiliations:** Department of Family Practice, Centre for Hip Health and Mobility, University of British Columbia, 2635 Laurel St, Vancouver, BC V5Z 1M9 Canada; University of Waterloo, 200 University Ave W, Waterloo, ON N2L 3G1 Canada; Centre for Hip Health and Mobility, University of British Columbia, 2635 Laurel St, Vancouver, BC V5Z 1M9 Canada; Department of Sociology, University of British Columbia, 6303 N.W. Marine Drive, Vancouver, BC V6T 1Z1 Canada

**Keywords:** Home health care, Home care, Family caregivers, Direct care workers, Informal/formal care partnerships, Domiciliary care, Caregiving

## Abstract

**Background:**

Health care discourse is replete with references to building partnerships between formal and informal care systems of support, particularly in community and home based health care. Little work has been done to examine the relationship between home health care workers and family caregivers of older clients. The purpose of this study is to examine home support workers’ (HSWs) perceptions of their interactions with their clients’ family members. The goal of this research is to improve client care and better connect formal and informal care systems.

**Methods:**

A qualitative study, using in-depth interviews was conducted with 118 home support workers in British Columbia, Canada. Framework analysis was used and a number of strategies were employed to ensure rigor including: memo writing and analysis meetings. Interviews were transcribed verbatim and sent to a professional transcription agency. Nvivo 10 software was used to manage the data.

**Results:**

Interactions between HSWs and family members are characterized in terms both of *complementary labour* (family members providing informational and instrumental support to HSWs), and *disrupted labour* (family members creating emotion work and additional instrumental work for HSWs). Two factors, the care plan and empathic awareness, further impact the relationship between HSWs and family caregivers.

**Conclusions:**

HSWs and family members work to support one another instrumentally and emotionally through interdependent interactions and empathic awareness. Organizational Care Plans that are too rigid or limited in their scope are key factors constraining interactions.

## Background

For many older people, receipt of home care services is the *only* option that enables them to remain independently and safely in their home. Between 2008 and 2011, Canadian home care clients increased by 55 %. At present, more than 1.4 million Canadians receive publicly funded home care services annually. As the populations continue to age and with earlier discharges from hospital, the need for home care [1] and associated costs will continue to escalate.

Often referred to as the ‘eyes and ears’ of home care [2], home support workers (HSWs) - also known as domiciliary, direct care or paraprofessional workers - provide assistance for older adults in the community with tasks such as bathing, dressing, medication use, toileting, and light household tasks [3]. Alongside receiving paid home care services, older adults are also likely to have the support of a family member, often referred to as a family caregiver [4]. It is well documented that older adults who do not have a family caregiver are at greater risk for institutionalization and that a critical step in supporting older adults in the community is to better understand the needs of their family caregivers [5].

In the last decade, there has been a notable shift in discourse towards the sharing of care between paid health-care professionals and unpaid family caregivers [6–8] and the promotion of partnerships between formal (paid) and informal (family) caregivers for older adults receiving home care. Together, HSWs and families provide the bulk of care for older adults living at home. Sims-Gould & Martin-Matthews [9] report that family members perceive that they ‘share the care’ with HSWs. However, the perspectives of HSWs about the family members of their older clients are less well known. Timonen notes that “formal and informal care occurs in tandem more prominently in the community context” ([10], p. 307), as in the delivery of home based care. In this study, we extend our understanding of home based care through an examination of HSWs perceptions of their older clients’ family members. In particular, we focus on whether HSWs view family members as helping and/or hindering them in the delivery of home based health care to older clients.

During a time of worker shortages and increased focus on recruitment and retention initiatives for HSWs [2], there is a need for an improved understanding of worker experiences, including their interactions with family members [4, 5, 11]. A better understanding of HSWs perspectives about family members will complement existing research about family caregivers’ perspectives of HSWs and HSWs’ perspectives of their clients.

### Home support workers perceptions of family

Most research about health care staff perceptions of families has been in the nursing literature or in residential care settings e.g., [6, 11, 12]. The provision of care in a residential care setting is very different than receiving care in the private space of one’s home. For example, each household is unique with respect to physical set-up, condition and sanitation. Compounding this, workers are expected to adjust daily to multiple homes (not just work within one environment as in residential care) encountering numerous clients, various family members and unpredictable interpersonal circumstances [13]. Unlike in residential care settings, HSWs do all of this in the absence of immediate, accessible supervision or staff support [14].

Moreover, existing research about relationship aspects of home support work typically focuses on interactions between clients and workers [10, 15–19]. Workers’ perceptions of family members are typically embedded in discussions of clients or of workers’ perspectives generally e.g., [15, 20] . To date, there are three studies that broadly address HSWs’ perceptions of family members of older adult clients [21–23]. Chichin [21] show that, for the most part, workers rated their experiences with families as positive (e.g., 80 % reported that families were very or somewhat helpful). The most common complaint was that families treated workers as maids; as well, workers’ job satisfaction declined when families expected the workers to go beyond what they perceived their job description to be in the home. Fischer and Eustis [22] describe three types of interactions between workers and families: caregiving alliances, conflict and separate worlds. Hokenstad and colleagues [23] explore HSWs’ perspectives about their interactions with family caregivers with a focus on understanding worker-family interactions when ending formal home care services. In such circumstances, workers display empathy for families and stress the importance of establishing clear boundaries. They also report that families are a critical source of information about clients. Even though attributes of relationships, such as companionship, trust and conflict, are identified as critical for partnerships between families and workers in health care settings e.g., [12, 24], this is an underdeveloped area of investigation in the home care literature.

In addition to describing the nature of the relationship between HSWs and family caregivers, we extend previous work and examine whether HSWs view family members as a help and/or a hindrance in the delivery of home based health care. Our work is guided by a socio-ecological approach [25] in that we pay particular attention to individual, organization and societal influences on HSW experiences of family members.

## Methods

This study is based on data from a larger mixed-methods study about home care delivery to older clients, involving home care managers, HSWs, older clients themselves and their family members [9, 13, 26, 27]. Qualitative and quantitative data was collected through in-depth semi-structured interviews. Drawing on the qualitative interview data, this paper focuses on the HSWs’ perceptions of clients’ family members.

### Setting and participants

Ethics approval was granted from the University of British Columbia Behavioural Research Ethics Board (BREB) and the Fraser Health Authority Ethics Board, the regional health authority in which our study was conducted. Data were collected as part of a study that ran from March 2007 to October 2012 in the Lower Mainland of British Columbia, Canada. Eligible HSWs were those able to participate in an English language interview and providing care for clients aged 65 and over through a home care agency. We also employed a second method of recruitment, identifying participants from a list of workers represented by the BC Government Employees Union (BCGEU local 403). Every fourth worker on the list was contacted. In all, 118 HSWs were interviewed: 84 (71 %) were recruited through the agency method and 35 (29 %) were recruited through the union (for additional recruitment information see: [13]). All of the HSWs we interviewed were unionized (Table 1 reports demographics). All of the participants in our study provided written informed consent. The findings are based on a total of 117 participants; one participant was excluded because she had to leave early and was not asked any of the family questions. One participant was interviewed via telephone at their request.

**Table 1:** Participant Characteristics

*N* = 117	Average	Range
Age	50	27-65
	Number	Percent
Gender		
Female	110	94.0 %
Education		
High School or less	35	30.0 %
Some College or University	20	17.0 %
Completed College or University	52	44.5 %
Registered Nurse	10	8.5 %
Place of Birth		
Canada	36	30.8 %
Philippines	36	30.8 %
Europe	10	8.5 %
Other	35	29.9 %
	Average	Range
Years Employed as HSW	12	<1- 29
Number of Clients Per Day	4	1-9
Work Status		
Casual	34	29.1 %
Part-time	11	9.4 %
Full-time	72	61.5 %

### Data collection and analysis

Interviewers used interview guides and probing techniques to increase the depth and quality of responses to the open-ended questions [28]. For this paper, our analysis focuses on data obtained when HSWs were asked about their interactions with and perspectives of clients’ family members (Table 2 contains interview questions). Interviews were conducted face-to-face at different locations (e.g., participant’s home, library) and ranged from 60 to 90 min. They were digitally recorded, transcribed verbatim and saved using ID numbers and pseudonyms. The pseudonyms are used within this manuscript. Nvivo 10 software was used to manage our data.

**Table 2:** Interview Questions

For those clients that have a spouse or partner, are they usually present when you are working in their home?
Do you have contact or reason to interact with him or her?
If so, what is that interaction like? Do you get along?
Do family members make your work more *difficult*? How?
Do family members make your work more *difficult*? How?
Do family members *help* your work? How?
In your opinion, in addition to the care that you provide, do family members also provide care to your older clients? If yes, explain.

Our analysis team consisted of post-doctoral fellows, a masters prepared research assistant and the principal investigators of the study, all experienced in qualitative research and analysis. Transcripts were reviewed using framework analysis, framework analysis is better adapted to research that has specific questions and a pre-designed sample. The prime concern is to describe and interpret what is happening in a particular setting, in this case the home care setting [29]. It is heavily based in, and driven by, the original accounts and observations of the people it is about, and it allows within-case and between-case analysis, it is comprehensive [30]. In the analysis, data is sifted, charted and sorted in accordance with key issues and themes using five steps: 1. familiarize; 2. identify a thematic framework; 3. index; 4. chart; 5. map and interpret [29, 30]. The following topic codes, alongside excerpts of data and preliminary definitions, were discussed during team analysis meetings: ‘HSWs help families’; ‘families help HSWs’; ‘families hinder HSWs’; ‘relational responses’; ‘respite’ and ‘strategies’. Through an inductive iterative process, using memo writing and analysis meetings, our team developed analytic codes that included: ‘interdependent interactions’, ‘strained interactions’, and ‘empathetic awareness’. Coding and final themes reported were developed through a process of collaboration and consensus amongst the authors via memos and analysis meetings.

Rigour was established through a combination of techniques that included a recording of decisions made throughout data collection and analysis (i.e., an audit trail), peer debriefing among members of the research team to promote reflexivity, via focused discussion about the developing themes, and extensive memo writing [31–33].

## Results and discussion

Most workers spoke passionately about their interactions with families, in both positive and negative terms. HSWs noted that family members can both help and hinder their care efforts; in some cases family members can do both simultaneously.

Helpful efforts included informational and instrumental support provided to workers from family members. HSWs underscored how families can provide them with *informational support* related to the care of their older clients and vice versa; they *complemented one another’s labour* by sharing vital techniques (e.g., for lifting, making transfers, etc.) necessary for safe care provision with families. As well, HSWs outlined the *complementary instrumental support* provided by families balanced by the additional ‘work’ often generated when family members were involved. However, where interactions were strained, in some cases, the family members *disrupted labour.* HSWs also explained how their work was hindered by the emotional ‘workload’ created or exacerbated by family members. We further discuss *complementary* and *disrupted* labor below. In addition, two additional factors will be discussed that mitigated the relationship between HSWs and family members – organizational service care plans which stipulated labour and empathetic awareness.

### Complementary labour: providing informational support

Families helped workers to provide care that took into account client routines and preferences by sharing information and expertise –families essentially ‘filled in’ knowledge for workers and compensated for information that they did not have or could not ascertain from clients. Melissa, a home care worker, explained how the family provided information that made it easier for her to provide quality care for the client:

… “Today, mom’s had a really rough time… she didn’t sleep all night long, so she’s going to be a lot weaker.” …So they’ll give you some advice that’s happened to them, which really helps us to go with our task.

In addition, families provided information about the home space that was critical to care provision, particularly during first visits to the home. Quinn explained:

*…they have to show you where things are ‘cause you’re going into a home blind. You need to know where the towels, all the bathroom stuff is… the cleaners… what belongs to the client, and what belongs to the rest of the family. Where the client sleeps, where the client eats. Is there special food just for the client…?*

Further, HSWs perceived themselves to be sources of both information and instruction for family members, and thereby compensated for what families did not know about caring for their relatives. For instance, workers taught families how to safely reposition and transfer their older relative, avoid bedsores, prevent spread of disease in the home and handle various medical conditions. Julie described a situation in which she taught the wife of a client how to better reposition her husband in a way that would decrease her chance of injury when doing transfers:

*They’re [family] trying to tell you how to get …them, out of the bed, especially a woman if you’re helping her husband. And I’ll say, “It’s okay. I know what I’m doing.” “But that’s not the way I do it.”…“No, but you have a sore back every day, don’t you?” “Well, yeah.” “Well, that’s because you’re not doing it right.” “Oh.”*

Although families sometimes doubted her, Julie was able to explain to them the benefits of her technique; in doing so she filled both an instructional and preventative role. HSWs also told families about agency or community services that they could access and provided ‘tips’ for caring for their family member, such as the use of larger incontinence pads and pill crushers. HSWs updated family members about clients’ needs, behaviors, and health status. HSWs perceived this role to be especially helpful for family members, and particularly for those adult children who did not live in the same home as the client:

*Sometimes they like to talk, “How you doing? What’s happening? Is everything okay? Does my mum need something? Just please let us know.” So… it’s really good to be close to the family. Because they want to know what’s happening … And you are there all the time.*

With their regular presence in the home, workers were positioned to report on changes in a client’s condition, and provided family members with information about their relative. As well, HSWs discussed how they ensured that their clients were receiving comprehensive care by updating families when a client was low on medication, supplies or groceries.

### Complementary labour: providing instrumental support

HSWs noted that families work to ensure that the home environment was an appropriate place to receive and provide care for their loved ones. They did this by purchasing supplies and ‘setting up’ prior to the arrival of the HSW. Gwyneth talked about how a family member provided extra assistance that facilitated her own work:

*One daughter definitely puts herself out a lot before she leaves to make sure that there’s veggies prepared ahead of time…to give you a head start because this client is very, very slow, right. So she’s very good in that aspect.*

Many HSWs talked about the importance of family members who purchased groceries and accompanied clients to appointments, tasks which HSWs could not typically do under the regulations of home support. As Noel noted, “yes they do [help], this daughter, she’s actually a doctor… they buy the food for the parent.*”*

In addition, families assisted with care activities that workers could not complete on their own, such as lifting or transferring heavy clients. They also helped HSWs to complete care tasks within the designated time frame, or finished workers’ tasks when out of time. For instance, Melissa described how families assisted her with set-up when working within tight timeframes:

*… you’re given an hour to do specific amount things and …if they’re in wheelchairs and you got to transfer them, you don’t have time maybe to get their clothes laid out…so their family member will have their clothes laid out… and they’ll have things ready.*

### Disrupted labour: creating additional instrumental work

Workers perceived that family members added to their instrumental workload by creating messes in the home, or by adding their dishes and laundry to those of the client so that the worker would have to also attend to them. Adding tasks beyond those prescribed for the client was predominately an issue in intergenerational households, where younger adults and children lived with the client. Katie explained:

*And sometimes family members want you to do things that you’re not allowed to do… I’ve been to a client’s house before…and the son wanted me to do his laundry… “actually, no, I’m not here for you,”…*

Several workers felt that families interfered with the plans they had for the time they spent with clients. Melissa explained:

*…sometimes they can mess things up for us. We might have already planned what we’re going to give them for their meals, and then they come in and they take it away, and it’s like, “Well, I thought I had that sandwich made and everything.” Now, I’ve got to redo the sandwich or something. So now they’ve really…messed our schedule up. They’ve messed things up.*

Carolyn also explained that in some circumstances, their workload was increased when families did not respond to requests for supplies:

*Some because, see, you need equipment like rubber gloves… to clean the bathroom or a mop or a broom…And they go, “Oh, yeah, yeah, yeah,” and you never see those things.*

### Disrupted labour: creating emotional work

Workers reported that in some cases, family members were disrespectful, rude and made them feel inadequate at their jobs. These strained type of interactions ranged from feelings of lack of respect for the workers’ role and/or time, to verbally abusive behaviour from families. Several workers preferred to work in some homes when family members were not present. Workers also commonly observed that family members made them feel ‘watched’ or monitored in their work. As Monica noted, “They just worry about that you are not the doing the best for [their] parents, so they just watch you very intense and they watch you, something like that or sometimes give you some order.*”*

Family members’ concerns were sometimes experienced by workers as criticism, with negative repercussions for all parties involved. A few workers reported that when they felt criticized or uncomfortable, they limited their interactions with the family, or rushed to avoid an unpleasant situation. For instance, Julie explained, “I really prefer them not to be there ‘cause they’re very critical. They know best… and you just kinda learn to turn the hearing aid off and just go about your business and you get it done and you get out.” In more contentious situations, workers were asked for proof of education, or engaged in disputes with family members regarding how care was provided. Care provided in the ‘home’ presented challenges for workers in terms of interactions with families who expected a level of knowledge about ‘each’ home and client that workers could not or did not always possess. Devon explained that it was difficult to remember ‘how’ everyone liked things done:

*… they prefer us to follow their way…the way to do the food, the way to put.. the way to clean… the washroom… have to…follow her way. So that’s quite…difficult…because we go to different clients every time, how can we remember different clients, different…way to work for them.*

Workers also described situations where they found themselves caught in the middle of family disputes and attending to family demands, expectations and conflicts which added an emotional layer of complexity to their workday.

### Mitigating factor: empathic awareness

Empathic awareness, an appreciation of the conflicting and negative emotions and feelings experienced by family members [34], was evident in our data as a factor that influenced interactions between workers and families. Workers expressed empathy for families, whom they understood to be just trying to do the best they could under the circumstances:

*If you go into a new situation and they see a different worker…some of them get nasty. “I’m, you know, I’m sorry that your other worker is on holidays but, you know, I’m here to provide a service,” and you try to explain to them that. But they build up a relationship with someone else and having someone new, like they just don’t like it sometimes.*

Several workers described the use of passive strategies, such as avoiding confrontation, ignoring issues or being flexible, in order to ease interactions with family members. Through their decisions to act passively in acknowledgment of the pressures that caregivers face HSWs demonstrated empathic awareness of these family situations:

*I’ve had family members where they go ballistic on you… But in the end, you just have to let them go through their little phase and then they’re fine, right? So some can be a little bit difficult at times. But it’s just that they get themselves worked up.*

Despite some of the challenging family behaviours encountered, workers were very aware of what the provision of care means to families especially the respite experienced by families during worker visits. Justine explained:

*Because it’s the only time they get sometimes to go out. One or two hours we provide them and that’s the only time they get, like, a husband who is looking after the wife and he only gets two hours when I’m there or somebody’s there. For that two hours they can do the-- go shopping or anything like that within that time.*

Several workers were sent to clients’ homes specifically to provide formal respite care; however many simply viewed their presence in the home as a form of respite for family caregivers. They often spoke of this help, using general terms such as ‘a break’ or ‘relief’. Furthermore, when workers had limited interactions with family members, it was often because family members were meant to be out of the home while HSWs were present, and thus gaining respite from their 24 h caregiving duties. Kristin explained*,* “We’re supposed to give them a break, you know…That’s the whole idea. They’re supposed to go out…” Thus, limited interactions between the workers and family members were not necessarily indicative of a negative situation; rather, some workers were insistent that because they are there for respite, the family should not be present. Workers were also careful to note that the family members’ ability to receive respite was predicated on rapport with and trust of the worker. Beyond making families feel comfortable, HSWs understood the important role that respite plays in supporting caregivers on an emotional or psychological level; they often spoke of relieving ‘burden’ and diffusing stress.

### Contextualizing factor: organizational service plans

The Care Plan is an organizational tool developed by the agency employing the HSW, to guide the scope of care tasks provided in the home. The Care Plan was frequently at the heart of disagreements between workers and families. Previous literature has identified that case managers, those who organize the overall Care Plan, often structure HSW’s role to be supplementary to informal, family caregivers [35]. However, in many situations, workers and family members were working at ‘odds’ because of an organizational plan for services to which workers were expected to adhere, but which families often felt was inadequate. In the majority of cases, conflicts arose due to disagreements about the scope of the care provided by home support services.

Workers frequently discussed how the Care Plan document framed their interactions with family members. Stella stated: “They will also sometimes add extra duties other than on the care plan. Then you gotta explain to them again, yeah.” As Stella observes, dealing with families’ expectations in relation to the care plan can be time consuming; many workers mentioned having to provide family members with multiple (re) explanations of the care plan. As well, workers expressed frustration about families asking them to perform housekeeping duties not listed in the Care Plan. Occasionally, requested tasks were not even legitimate functions in HSWs’ job descriptions (e.g., vacuuming and dusting or more demanding jobs, such as cleaning the attic). Justine explained:

*… sometimes you feel like that they’re too nosey and they are trying to make you do more work, more than you are supposed to be doing…They think that we are their maids…Like they want us to do cleaning the windows and do everything, do the dishes for the whole family and everything.*

Workers reported that they contact their supervisors or nursing managers in response to conflicts with family members, thereby seeking intervention from agency staff to mitigate a difficult situation with clients’ relatives. Workers also invoked the assistance of their employing agency when they were unsuccessful in explaining a policy to a family member or needed advice on how to deal with challenging situations. Lydia explained how she dealt with situations where families wanted her to go above and beyond the Care Plan: “You’ve just got to try and handle it as best you can for that day and then phone the supervisor*.”*

## Conclusions

We explored HSW-family interactions in home care from the perspective of workers. Corroborating research findings in care settings such as assisted living and nursing homes [36], we found both positive and negative aspects to these interactions e.g., ‘[11]. In most cases, family members played a role in shaping the quality of the HSWs’ work environment. While our results demonstrate that families helped by maintaining the home and thereby the work environment, we also found that families occasionally created additional work for HSWs. Encounters between HSWs and family members are highly variable, ranging from entering one home to provide respite for an exhausted caregiver to, in the next home, being confronted by a family critical of the worker’s approach to meal preparation. Workers in our study visited, on average, four clients per day, thus requiring them to adapt their approach to clients and to families from one visit to the next.

Even though the client in home care is the older person, the work and negotiations around care provision very much involve the family. This involvement can be interdependent and provide a two way system of support for worker and family member. In this way, workers and family members are working together as allies in care, engaged in complementary labour [22]. On the other hand, their engagement can be strained. Workers reported feeling watched and taken advantage of by family members; such experiences have direct implications for job satisfaction and job tenure in the long term [37]. In extreme circumstances workers felt violated and abused. HSWs conduct their work in what is traditionally thought of as the domain of the family - the private sphere of the home. Working with families in intimate ‘home space’ required interpersonal skills and sensitivity. Where the interactions with family are negative, the work is more difficult. This has implications for retention of workers who identify the interpersonal aspects of their work as key to the reasons they like their job [27].

HSWs discussed how family members helped them with the tight timelines that they are allotted, often only 50 min per client [38]. Family members helped with time pressures by setting-up, keeping the home tidy, assisting with caregiving tasks and orienting the worker to the space. Within a context of worker shortages [39] and funding cutbacks [40], it is both frustrating for workers and inefficient for the system when HSWs must spend time defending the scope of care, rather than providing direct care to clients.

Workers were, for the most part, very empathetic towards family members and recognized their unspoken role in providing family caregiver support. Similarly, family members deeply value the contributions made by HSWs [9, 13]. It has been previously suggested that HSWs need to recognize family caregivers as valued coworkers, and be more perceptive of the family members’ individual needs in order to optimize the effectiveness of care [8]. However, our findings refute this notion. Evidence from this study suggests that many HSWs acknowledge the reciprocal partnership that should ideally exist between formal and informal caregivers, and act on that basis. Workers recognized family stress, burden and frustrations [18, 22]. Interventions to foster empathy between staff and clients’ relatives have been developed in facility-based care [e.g., 41], and are certainly relevant in home care settings.

Many HSWs were emphatic that family members require respite from their caregiving duties. Researchers have noted the ethical dilemma inherent in advocating for partnerships with families to provide care, as it can lead to increased expectations and exploitation of the relatives [20, 41]. However, our research with HSWs suggests that workers do not seek to further exhaust or exploit family members; rather, they view their role as doing precisely the opposite. Case managers often outline the role of HSWs as supplementary to informal family care in order to promote the sustainability of the support system in the long-term [35]. Our research indicates that many HSWs subscribe to this supplementary role, and often seek to provide support not only to the client, but also to family members within the care network.

Our analyses of home support worker and family data illustrate how individual parties are constrained by the broader health care system. Workers and family members are both ‘under pressure’ based on restrictive Care Plans, unmet needs and agency policies. As a result, family members [9, 13] frequently expressed a desire for ‘task substitution’ (i.e. asking workers to sit and have a cup of tea with their relative, rather than those outlined in the Care Plan). Workers attempt to balance the needs and preferences of clients and family members with the contractual obligation outlined in the Care Plan. A worker who deviates from the Care Pan is subject to disciplinary action. In our study, several workers expressed frustration when task substitution is expected or demanded by family members. This again places workers in a difficult situations requiring them to balance the satisfaction of family members, and their empathy for family members, against the tasks that they are contracted to provide.

### Individual and organizational implications

HSWs depend on the knowledge of families to provide personalized care in older client’s home settings, something they know is important to families [9], and to home care clients [26]. In order to optimize care, home care agencies should endeavour to support and sustain positive working relationships between workers and families by facilitating clear and shared expectations regarding care and the scope of work provided. This could be done through traditional case management conferences or through in-service training sessions involving families, workers and managers. The natural partnership that is possible between families should be acknowledged and supported. In doing so, with additional training, shared understanding of the scope of the HSWS role and clear mechanisms for communication and conflict resolution, many of the issues faced by workers (and families) could be alleviated.

Our findings underscore HSWs’ relational competence – the ability to empathize with the situations of others and to respect the work they do [42]. Specifically, HSWs demonstrated a deep understanding of home support as respite from the care that family members provide. The ability to tailor care to the needs of their clients and families, is critical to the development of partnership models of care [43]. As such, when Care Plans are too structured, there is little flexibility for HSWs and family members to make decisions about care. Care collaborations are generally considered to be relationships that “unfold over time within the context of and in response to multi-level factors” ([5], p. 30).

### Limitations

A limitation of this paper is that family members’ perspectives are not presented in these analyses. Where appropriate, we have compared and contrasted these findings to results as reported in an earlier publication about families’ perspectives of HSWs [9]. Future research based on matched pairs of HSWs and family members linked to the same client will further extend this line of enquiry. In addition, we recommend an observational research design that allows for an exploration of what workers do, not just what they say they do, during interactions with family members. We also limited our sample to those HSWs that could participate in an English interview, it may be that those with less proficiency in English would have a different, more marginalized experience. Again, an observational study would be of benefit for those who are less proficient in English.

## Abbreviations

BCGEU: BC Government Employees Union
BREB: University of British Columbia Behavioural Research Ethics Board
HSW: home support worker
SD: standard deviation
